# Comment on ‘A Causal Effect of Serum 25(OH)D Level on Appendicular Muscle Mass: Evidence From NHANES Data and Mendelian Randomization Analyses’ by Ren et al.—The Authors' Reply

**DOI:** 10.1002/jcsm.70067

**Published:** 2025-09-23

**Authors:** Qian Ren, Jinrong Liang, Yanmei Su, Ruijing Tian, Junxian Wu, Sheng Ge, Peizhan Chen

**Affiliations:** ^1^ Department of Clinical Nutrition Shanghai Sixth People's Hospital Affiliated to Shanghai Jiao Tong University School of Medicine Shanghai China; ^2^ Department of Oncology Shanghai Sixth People's Hospital Affiliated to Shanghai Jiao Tong University School of Medicine Shanghai China; ^3^ Department of Endocrinology and Metabolism, Shanghai General Hospital Shanghai Jiao Tong University School of Medicine Shanghai China; ^4^ Clinical Research Center, Ruijin Hospital Shanghai Jiao Tong University School of Medicine Shanghai China

We would like to thank Zeng et al. [1] and Zheng et al. [2] for their comments on our recently published manuscript suggesting that serum 25(OH)D level may be causally associated with the appendicular muscle mass (AMM) [3].

We concur with Zeng et al. [1] and Zheng et al. [2] that sample overlap in Mendelian Randomization (MR) analyses may introduce potential bias and increase type I error rates, a concern that has been extensively documented in methodological studies of MR [4, 5]. Nowadays, genome‐wide association studies (GWASs) consortium usually integrate numerous large datasets for meta‐analysis [4]. In two‐sample MR studies, complete sample non‐overlap cannot always be guaranteed, as large GWAS consortium summary statistics are now commonly employed as both instrumental variables for exposures and for outcome associations in MR analyses [4]. We noticed that recently published two‐sample MR studies typically had a high or low proportion of overlapped samples even in these well‐designed investigations [6, 7, 8]. In our study, we noticed a significant association between serum 25(OH)D level and AMM in the NHANES dataset with a large sample size (*n* = 11 242) [3]. We would like to assess whether there is a causal relationship between 25(OH)D level and AMM. Revez et al. performed the GWAS on serum 25(OH)D levels in UK Biobank participants (*n* = 417 580), identifying 143 independent loci that were significantly associated with serum 25(OH)D [9]. This study, which provided the most complete data in the IEU OpenGWAS database (ebi‐a‐GCST90000617), was used to extract IVs for serum 25(OH)D level in MR studies. To our best knowledge, only one large‐scale GWAS on AMM (*n* = 450 243) has been conducted to date, performed by Pei et al. [10] in UK Biobank participants. Using summary statistics from these two GWASs on serum 25(OH)D and AMM, we conducted the two‐sample MR analysis to assess their causal relationship, though the sample overlap was unavoidable.

According to recommendation by Zeng et al. [1], we assessed the magnitude of bias and control for inflated type I error rates using the online tools (https://sb452.shinyapps.io/overlap) as previously described [5]. We found the bias was estimated to be 0.001 with the type I error rate being 0.05, suggesting minimal influences caused by sample overlap in the MR studies. We also applied the MRlap method, which was designed to correct the joint biases caused by the Winner's curse, to assess the causal association between serum 25(OH)D level and AMM [4]. The MRlap algorithm calculates a test statistic to evaluate whether the corrected effect estimate is significantly different from the inverse variance weighted (IVW)‐MR observed effect. If there is no significant difference, the observed IVW‐MR estimate could be safely used to assess the causal associations between exposure and outcome. Based on the GWAS summary statistics for serum 25(OH)D (ebi‐a‐GCST90000617) [9] and AMM (ebi‐a‐GCST90000025 for all participants, ebi‐a‐GCST90000026 for male and ebi‐a‐GCST90000027 for female) [10] in UK Biobank participants, we assessed the causal estimate for serum 25(OH)D on AMM with the MRlap algorithm (MR_threshold = 5 × 10^−8^, MR_pruning_LD = 0.01 and MR_pruning_dist = 5000 kb). We noticed the observed effect was 0.0403 (SE = 0.0198, *p* value = 0.0421) for all participants and no significant inflation of the estimate was caused by sample overlap (corrected effect = 0.0421, SE = 0.0213, *p* value = 0.0478; *p* difference = 0.435). In the stratification analyses by gender, we noticed the observed effect was 0.0492 (SE = 0.0217, *p* value = 0.0236; corrected effect = 0.0495, SE = 0.0232, *p* value = 0.0331; *p* difference = 0.920) for male and 0.0354 (SE = 0.0222, *p* value = 0.111; corrected effect = 0.0387, SE = 0.0235, *p* value = 0.0991; *p* difference = 0.454) for female. These results were consistent with our previously published two‐sample MR studies [2], indicating minimal influences on the causality estimates caused by sample overlap.

Zheng et al. pointed out that several SNPs (e.g., rs1047891, rs11076175, rs11204743, rs1260326, rs2756119, rs4616820, rs512083, rs55707527, rs55872725, rs6011153, rs72862854, rs7528419, rs7864910, rs804281 and rs8107974) are significantly associated with AMM [2], which may lead to the pleiotropy effects in two‐sample MR studies [3]. In our study, we have performed the leave‐one‐out analyses and found no individual IVs significantly influenced the overall estimates in our MR studies [3]. After excluding these SNPs that were significantly associated with AMM (*p* < 1 × 10^−7^) and potential palindromic SNPs, the fixed‐effects IVW model also suggested that genetically higher serum 25(OH)D levels were positively associated with AMM in all participants (*β* = 0.016, SE = 0.008, *p* = 0.044) and males (*β* = 0.036, SE = 0.012, *p* = 0.002) but not in females (*β* = 0.014, SE = 0.010, *p* = 0.177). To exclude potential pleiotropy effects of these genetic IVs, we also applied the MR‐Egger bootstrap regression method, which provided more robust results in the presence of pleiotropy or weak instruments than the MR‐Egger regression test [11]. The estimated effect of serum 25(OH)D on AMM was 0.032 (SE = 0.014, *p* = 0.012) for all participants, 0.039 (SE = 0.021, *p* = 0.031) for males and 0.028 (SE = 0.019, *p* = 0.063) for females, according to the MR‐Egger bootstrap regression tests. These results demonstrated that the main findings remain valid when these potentially pleiotropic variants were excluded in MR analyses.

Our gender‐stratified analyses revealed that the association between blood 25(OH)D levels and AMM was more pronounced in males than in females [3]. Conducting MR analyses using gender‐specific summary statistics would be preferable, as suggested by Zheng et al. [2]. However, the results from GWAS performed by Revez et al. suggested that gender was not significantly associated with serum 25(OH)D level in the European UKB participants, and gender‐stratified analyses were not performed in that study [9]. We performed the gender‐stratified MR analyses based on the summary statistics of the GWAS on AMM and found the causal effect size of serum 25(OH)D on AMM was 0.057 in males compared to 0.043 in females, which means that for a 1‐unit increase in log‐transformed vitamin D levels, the genetically predicted increase in AMM is 0.057 kg for males and 0.043 kg for females. Whether vitamin D supplementation exerts differential effects on AMM between sexes warrants further investigation through well‐designed randomized controlled trials.

Dual‐energy X‐ray absorptiometry (DXA) screening was conducted among individuals aged 8–59 years, as documented in the NHANES 2011–2018 data [3]. However, the present study focused specifically on a subset of this population—adults aged 18–59 years—for all analyses. Therefore, the reference to the age range of 8–59 years in the original text was intended to describe the broader scope of the NHANES dataset, rather than the specific age range of participants included in our study [3].

Again, we thank Zeng et al. [1] and Zheng et al. [2] for their comments, which underscore the importance of addressing Winner's curse and pleiotropy effects in MR analyses of serum 25(OH)D and AMM [3]. Further well‐designed intervention studies are required to establish that vitamin D supplementation could improve the AMM in general populations.

## Conflicts of Interest

The authors declare no conflicts of interest.
